# Combined clinical variable and radiomics of post-treatment total body scan for prediction of successful I-131 ablation in low-risk papillary thyroid carcinoma patients

**DOI:** 10.1038/s41598-024-55755-6

**Published:** 2024-02-29

**Authors:** Maythinee Chantadisai, Jirarot Wongwijitsook, Napat Ritlumlert, Yothin Rakvongthai

**Affiliations:** 1 Division of Nuclear Medicine, Department of Radiology, Faculty of Medicine, Chulalongkorn University, Bangkok, Thailand; 2 Division of Nuclear Medicine, Department of Radiology, King Chulalongkorn Memorial Hospital, The Thai Red Cross Society, Bangkok, Thailand; 3 Division of Nuclear Medicine, Department of Radiology, Surin Hospital, Surin, Thailand; 4 Chulalongkorn University Biomedical Imaging Group, Department of Radiology, Faculty of Medicine, Chulalongkorn University, Bangkok, Thailand; 5 Biomedical Engineering Program, Faculty of Engineering, Chulalongkorn University, Bangkok, Thailand; 6 School of Radiological Technology, Faculty of Health Science Technology, Chulabhorn Royal Academy, Bangkok, Thailand

**Keywords:** Radiomics, Artificial intelligence, Thyroid cancer, Total body scan, Successful ablation, Biomarkers, Diseases, Endocrinology, Health care, Medical research, Molecular medicine, Oncology, Mathematics and computing

## Abstract

To explore the feasibility of combined radiomics of post-treatment I-131 total body scan (TBS) and clinical parameter to predict successful ablation in low-risk papillary thyroid carcinoma (PTC) patients. Data of low-risk PTC patients who underwent total/near total thyroidectomy and I-131 ablation 30 mCi between April 2015 and July 2021 were retrospectively reviewed. The clinical factors studied included age, sex, and pre-ablative serum thyroglobulin (Tg). Radiomic features were extracted via PyRadiomics, and radiomic feature selection was performed. The predictive performance for successful ablation of the clinical parameter, radiomic, and combined models (radiomics combined with clinical parameter) was calculated using the area under the receiver operating characteristic curve (AUC). One hundred and thirty patients were included. Successful ablation was achieved in 77 patients (59.2%). The mean pre-ablative Tg in the unsuccessful group (15.50 ± 18.04 ng/ml) was statistically significantly higher than those in the successful ablation group (7.12 ± 7.15 ng/ml). The clinical parameter, radiomic, and combined models produced AUCs of 0.66, 0.77, and 0.87 in the training sets, and 0.65, 0.69, and 0.78 in the validation sets, respectively. The combined model produced a significantly higher AUC than that of the clinical parameter (p < 0.05). Radiomic analysis of the post-treatment TBS combined with pre-ablative serum Tg showed a significant improvement in the predictive performance of successful ablation in low-risk PTC patients compared to the use of clinical parameter alone.

**Thai Clinical Trials Registry **TCTR identification number is TCTR20230816004 (https://www.thaiclinicaltrials.org/show/TCTR20230816004).

## Introduction

Thyroid cancer is the most common malignancy of the endocrine system, with a prominent gender disparity with a ratio between women and men that exceeds 3:1. Thyroid cancer is the seventh most common cancer in women in the United States, and occupies the fourth rank by prevalence and the seventh rank by incidence in Thailand^1^. The rate of thyroid cancer in Thailand is not different from the average rates in Asia and around the world, with the predominance of papillary thyroid carcinoma (PTC) in more than 70% of all thyroid cancer subtypes^2^. According to National Cancer Institute data, the rate of new cases of thyroid cancer was 13.9 per 100,000 men and women per year in 2016–2020^3^.

After total/near-total thyroidectomy in most cases, I-131 ablation/treatment leads to a significantly improved patient’s prognosis^4^. Successful remnant ablation is associated with better disease-free and overall survival, a lower rate of distant metastases, and a reduction in cancer mortality rates compared to surgery alone, and also helps in long-term follow-up of differentiated thyroid cancer (DTC) patients^5^. Currently, the most significant indicator for predicting successful ablation is the pre-ablative serum thyroglobulin (Tg)^4,6,7^.

The integration of artificial intelligence (AI) in several sectors, including healthcare, has grown exponentially worldwide in recent years^8,9^. Radiomics, an AI closely related field, is a quantitative approach to medical imaging, which aims at enhancing the existing data available to clinicians by means of advanced mathematical analysis^10–12^. This analysis is mainly applied to extract and analyze imaging features from various types of medical images and served as an imaging biomarker to determine association with clinical outcomes such as treatment responses and patient prognosis in several tumor entities^13–34^.

In recent years, there has been an increase in radiomic studies in thyroid diseases with various applications of ultrasound radiomics, from predicting malignancy in thyroid nodules^35^, prediction of lymph node metastasis in patients with PTC^36–38^ to the association between radiomic signatures and disease-free survival in PTC^39^. The use of radiomics to improve risk stratification in F-18 FDG-avid thyroid incidentalomas is also evidenced^40,41^. Therefore, the radiomic analysis of planar I-131 total body scan (TBS) to predict the patient’s outcome is interesting to explore.

This study aimed to explore the feasibility of using radiomic signature derived from post-treatment I-131 TBS combined with clinical parameters to predict successful remnant ablation in patients with low-risk PTC. If feasible, further research on the use of radiomics analysis in pre-treatment TBS or thyroid scan to help adjusting the treatment plan in low to intermediate-risk PTC is thought-provoking.

## Materials and methods

### Data collection

The study was approved by the Institutional Review Board of the Faculty of Medicine, Chulalongkorn University (COA No. 0873/2022, IRB No. 321/64). Data were retrospectively collected since July 2021. Low-risk PTC was defined according to the ATA 2009 risk stratification system with proposed modification^42^. Inclusion criteria were low-risk PTC patients who underwent total/near total thyroidectomy and 30 mCi of I-131 ablation, without RAI-avid metastatic foci outside the thyroid bed on the post-treatment TBS. All TBS were acquired at King Chulalongkorn Memorial Hospital (KCMH), Bangkok, Thailand, using the same protocol with matrix size of 256 $\times$ 256 and a 5-min acquisition using high energy collimator. Patients with serum anti-Tg levels greater than 100 IU/ml^6^ at the pre-ablative period or during follow up, patients who lost follow up at 6–12 months, or patients whose post-treatment TBS was unable to define region of interest (ROI) by automated segmentation were excluded.

### Clinical features

Clinical characteristics including age, sex, pre-ablative serum Tg, follow-up image data, and ablative outcome were collected. Successful ablation was defined when the follow-up result at 6–12 months met one of the following criteria: (a) negative I-131 TBS and stimulated Tg (sTg) less than 1 ng/ml, (b) no evidence of disease on neck ultrasound and suppressed Tg less than 0.2 ng/ml, and (c) 6-month neck ultrasound showed non-specific findings with no significant change or resolution when follow-up, and suppressed Tg less than 0.2 ng/ml^42^. The study design was prepared according to the recommendations in the guideline^43^.

### Radiomic features

**Figure 1:**
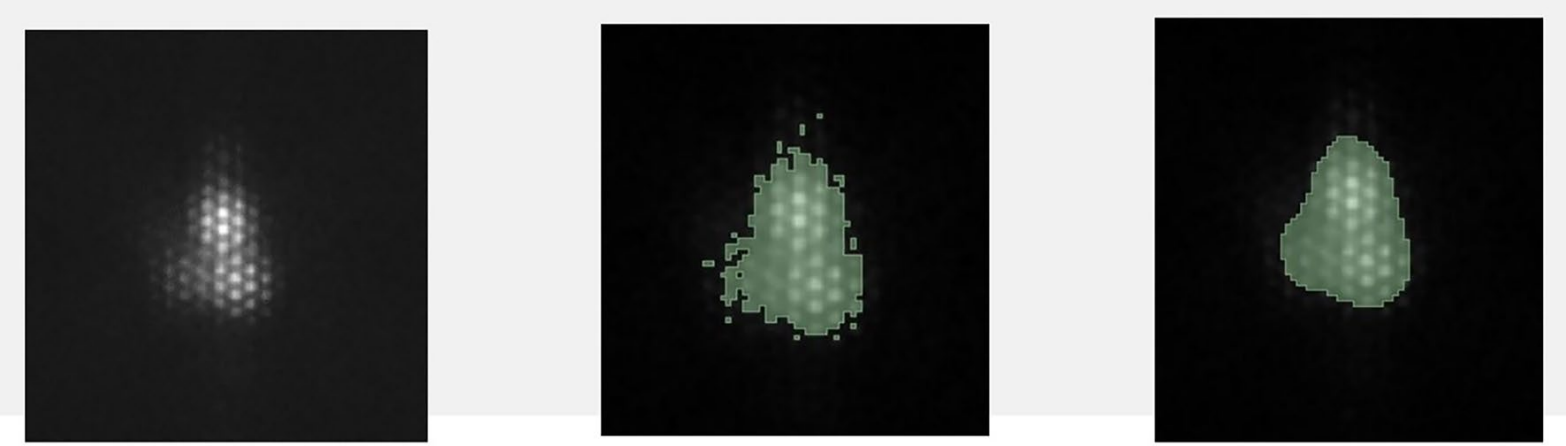
Automated image segmentation via 3D Slicer software.

**Figure 2:**
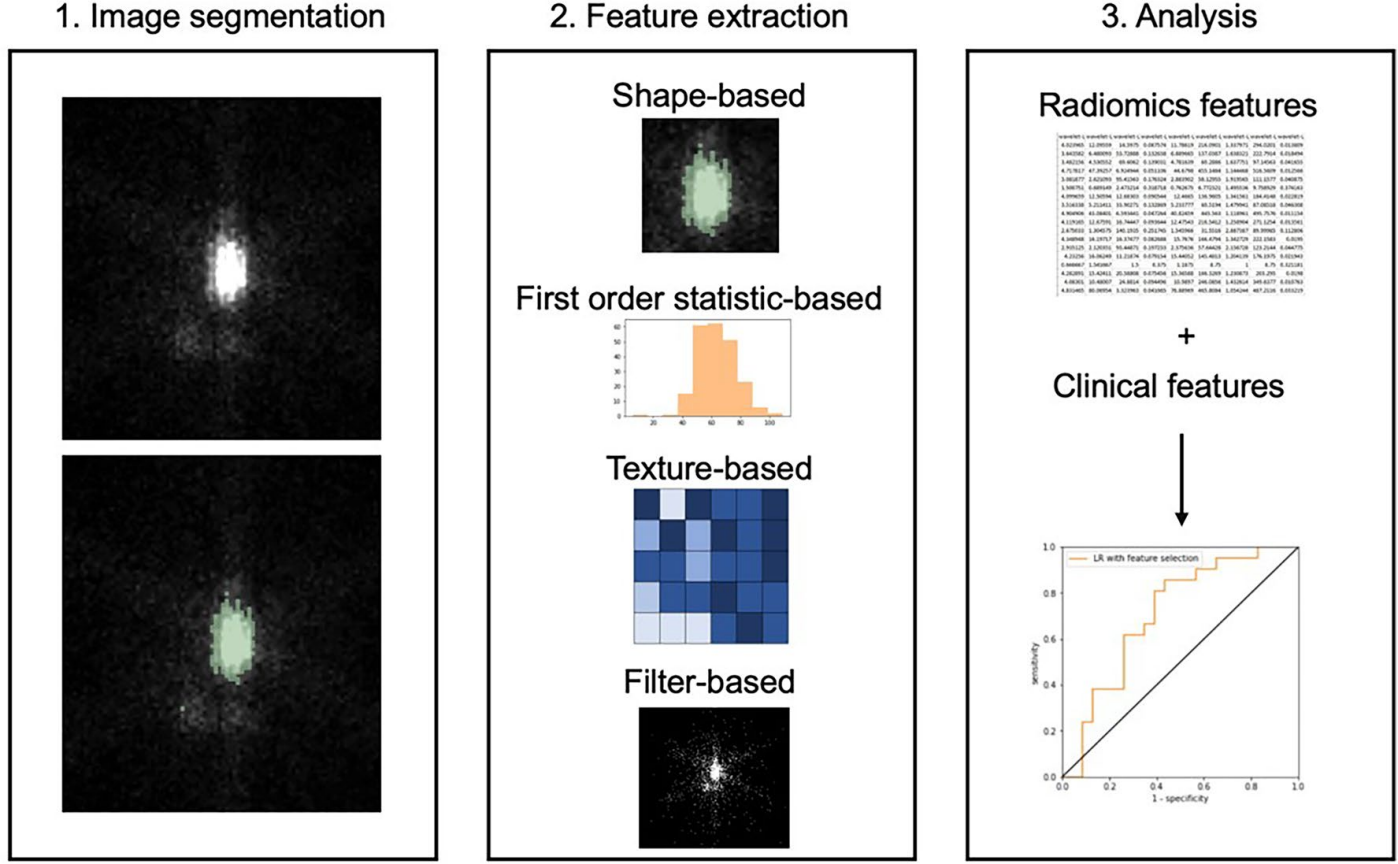
Radiomics workflow (1) image segmentation (2) radiomic feature extraction (3) analyze association of radiomic feature and clinical data.

The post-treatment I-131 TBSs were performed at 5–7 days after receiving I-131 and the radiomic extraction was performed from the post-treatment TBS in all patients. Automated segmentation was applied on the static anterior thyroid image of the post-treatment TBS using the 3D Slicer software version 4.10.2^44^ by using the threshold method with maximum entropy. Images were smoothed by the closing method (fill holes) using a kernel size of 8.0 mm and the Gaussian method using a SD of 3.0 mm (Fig. 1). Radiomic features were extracted into four classes: shape-based class, first-order statistics class, texture-based class, and filtered-based class, using PyRadiomics^45^ open-source software, as shown in the workflow (Fig. 2). For the radiomic model to operate at its best, the bin width parameter was varied between 0.4, 1, and 2. In the case of patients who had more than one ROI, we used the average value of radiomic features of all ROIs for the analysis.

### Predictive model construction

In model development, logistic regression with regularizations (Lasso and Ridge) was used with inverse strength ranging from C = 0.001 to C = 10 via Python from the scikit-learn library^46^. All radiomic features extracted from PyRadiomics were processed using the recursive feature elimination method to select the useful radiomic features for the prediction of successful ablation in radiomic model. The best hyperparameters were validated by fivefold cross-validation to select the best model performance based on the receiver operating characteristic curve (AUC). For the combined model, the significant clinical parameters were used as the variables together with all other radiomic features, and then a recursive feature elimination algorithm was applied to remove irrelevant features that did not significantly affect the predictive power.

### Statistical analysis

Descriptive quantitative data were expressed as means and standard deviations. An independent two-sample Student’s t-test was conducted to compare the data. Categorical variables are shown as percentages, and a chi-square analysis was applied to compare the results. A Wilcoxon signed rank test was used to compare the model performance of clinical, radiomic, and combined models. Statistical analyses were performed using IBM SPSS Statistics 22 software, and a *p*-value < 0.05 indicated a significant difference.

### Ethics approval

This study was performed in line with the principles of the Declaration of Helsinki. Approval was granted by the Ethics Committee of Chulalongkorn University (Date 21 June 2022/ COA No. 886/2021).

### Consent to participate

The Ethics Committee of Chulalongkorn University has given a waiver of informed consent due to the retrospective nature of the study in the manuscript.

## Results

### Patient characteristics

From April 2015 to July 2021, a total of 192 patients met the inclusion criteria. Among them, 35 patients were excluded due to high serum anti-Tg. Twenty-four patients were excluded due to the lack of follow-up data and three patients were excluded because of the inability to perform automated image segmentation. As a result, 130 patients were enrolled in our study.

The mean age of the patients was 45.56 (± 14.07) years, and 83.8% of them were women. The mean pre-ablative serum Tg was 10.54 (± 13.36) ng/ml. Eighty-five patients (65.4%) were followed up with diagnostic TBS and 45 patients (34.6%) were followed up with neck ultrasound at 6 months after I-131 ablation to evaluate successful ablation. The median follow-up time was 252 days. Of 130 patients, successful ablation was achieved in 77 patients (59.2%) and 53 patients (40.8%) did not meet the criteria of successful ablation. Most of the patients (113 patients, 86.9%) had a single segment of ROI, however, 16 patients had two ROIs, and 1 patient had three ROIs. The patient characteristics were summarized in Table 1.

**Table 1:** Demographic and clinical characteristics of patients.

Characteristics	Value
Patient, *n*	130
Age, years (mean ± SD)	45.56 (± 14.07)
Sex, n (%)	
Male	21 (16.2%)
Female	109 (83.8%)
Pre-ablation serum Tg, ng/mL	
Mean ± SD	10.54 (± 13.36)
Range	0.04–88.90
Follow up imaging, n (%)	
I-131 total body scan	85 (65.4%)
Neck ultrasonography	45 (34.6%)
Ablation outcome, n (%)	
Success	77 (59.2%)
Unsuccess	53 (40.8%)
Number of ROI segment, n (%)	
One	113 (86.9%)
Two	16 (12.3%)
Three	1 (0.77%)

The mean pre-ablative serum Tg in the unsuccessful group (15.50 ± 18.04 ng/ml) was significantly higher than that in the successful ablation group (7.12 ± 7.15 ng/mL) (*p* < 0.05). Neither the sex nor age of the patient at the time of ablation showed a significant association with the ablative outcome as shown in Table 2, thus the pre-ablative serum Tg was used to represent a significant clinical parameter for outcome prediction. Regarding the performance in predicting successful ablation, the clinical parameter yielded the AUCs of 0.66 ± 0.02 and 0.65 ± 0.11 on the training and validation sets, respectively, given its accuracy of 0.65 ± 0.07, and specificity of 0.32 ± 0.12.

**Table 2:** Result of univariate analysis for clinical factor affecting the outcome of ^131^I ablation.

Characteristics	Successful ablation 77 (59.2%)	Unsuccessful ablation 53 (40.8%)	P value
Age, years (mean ± SD)	46.05 ± 15.05	44.85 ± 12.62	0.258
Sex, n (%)			
Male	12 (57.1%)	9 (42.9%)	0.832
Female	65 (59.6%)	44 (40.4%)	
Pre-ablation Tg, ng/mL			
Mean ± SD	7.12 ± 7.15	15.50 ± 18.04	< 0.05
Range	0.04–34.80	0.04–88.90	

### Radiomics analysis

There was a total of 464 features extracted. By using the recursive feature elimination method, the significant radiomic features for prediction of successful ablation in the radiomic model were wavelet-LH_glrlm_RunLengthNonUniformityNormalized and wavelet-LH_glrlm_RunVariance. The radiomic model yielded the AUCs of 0.77 ± 0.02 and 0.69 ± 0.11 on the training and validation sets, respectively. There was no significant difference in AUCs observed between the radiomic model and clinical parameter, as shown in Table 3.

**Table 3:** AUC in train and validation set (repeat run 20 times).

Model type	Train AUC	Validation AUC	p-value*	No. of features
Clinical	0.66 ± 0.02	0.65 ± 0.11	–	1
Radiomics	0.77 ± 0.02	0.69 ± 0.11	0.229	7
Clinical + radiomics	0.87 ± 0.02	0.78 ± 0.10	0.006	10

*Compared with clinical model.

**Figure 3:**
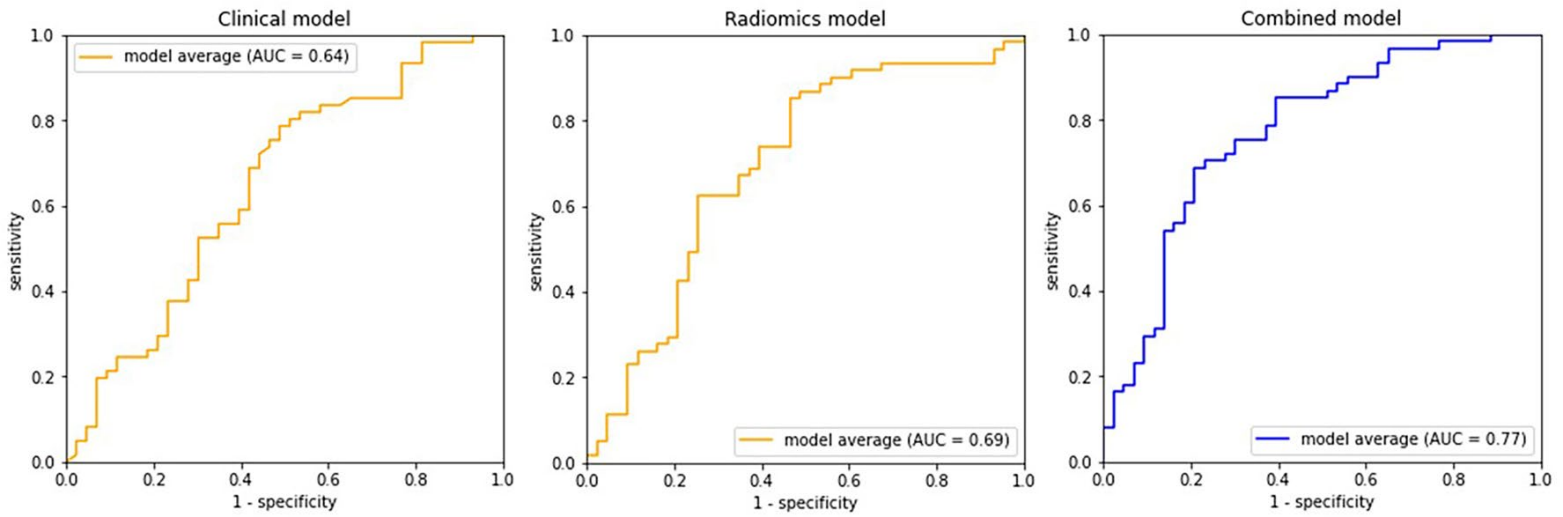
ROC curve analysis of clinical, radiomics and combined models to predict successful ablation. The AUC values in validation group were shown.

By adding radiomics to the clinical parameter, called the combined model, the AUCs were statistically significant higher compared to clinical parameter alone, with the AUCs of 0.87 ± 0.02 and 0.78 ± 0.10 in the training and validation sets, orderly. The ROC curves of radiomic model, clinical parameter, and combined model were plotted to show the performance in predicting successful ablation (Fig. 3). When compared to other models, the combined model showed better performance in almost all metrics with the accuracy, precision, F1-scores, sensitivity, and specificity of 0.74 ± 0.09, 0.76 ± 0.07, 0.78 ± 0.08, 0.81 ± 0.12, and 0.62 ± 0.12, respectively (Table 4). The combined model improved most of the predictive performance compared to the clinical parameter alone, especially in the specificity aspect.

**Table 4:** Model performance in validation set.

Model type	Accuracy	Precision	F1-score	Recall/sensitivity	Specificity
Clinical	0.65 ± 0.07	0.66 ± 0.05	0.74 ± 0.05	0.85 ± 0.08	0.32 ± 0.12
Radiomics	0.66 ± 0.07	0.69 ± 0.06	0.73 ± 0.05	0.79 ± 0.08	0.46 ± 0.15
Clinical + radiomics	0.74 ± 0.09	0.76 ± 0.07	0.78 ± 0.08	0.81 ± 0.12	0.62 ± 0.12

In addition to pre-ablative serum Tg, the significant radiomic features for the prediction of successful ablation in the combined model were as follows.

1. wavelet-HL_firstorder_Maximum
2. wavelet-HL_glszm_ZoneVariance
3. original_glcm_InverseVariance
4. wavelet-HL_glcm_DifferenceEntropy
5. wavelet-HH_gldm_DependenceVariance
6. wavelet-HH_glrlm_RunVariance
7. wavelet-LH_glrlm_RunLengthNonUniformityNormalized
8. wavelet-HL_glcm_InverseVariance
9. wavelet-HL_glcm_ClusterProminence

## Discussion

Our results showed that the addition of radiomics to the clinical parameter, in which we called combined model, improved the predictive performance of successful I-131 ablation when compared to the use of the clinical parameter alone. The gaining value of the predictive performance of the combined model is obviously demonstrated especially in the specificity aspect. The pre-ablative serum Tg is widely validated as a significant clinical parameter for the prediction of successful ablation, which was also demonstrated in our study. High pre-ablative serum Tg levels correlated with unsuccessful ablative outcome (*p* < 0.05) with the AUC of 0.65 in our study, corresponding to the data from the previous studies of AUCs of 0.620–0.917^4,6,7,47^. However, the predictive value of pre-ablative Tg is undoubtedly diminished by the interference of the large amount of the residual tissue after surgery^48^, which may limit its use in some cases. The mean pre-ablative Tg in our study was slightly higher than the data in other studies, which probably represents larger residual thyroid remnant in the absence of I-131-avid metastasis from our data in the post-treatment TBS^47^.

Furthermore, the negative pre-ablative Tc-99 m pertechnetate scan and the Tc-99 m pertechnetate uptake rate value < 0.9% were also the significant predictors of successful ablation with AUC of 0.710 as demonstrated by Giovanella et al.^47^. Nevertheless, the addition of pre-ablative pertechnetate scan requires additional patient-related imaging procedure and time, and further validation of the tests in higher pre-ablative serum Tg subgroup is still warranted.

The combined model improved the predictive performance of successful I-131 ablation in almost all aspects, including the accuracy, precision, and evidently improved specificity compared to the use of pre-ablative Tg alone. To our knowledge, this was the first study to use a radiomic approach in the post-treatment I-131 TBS to predict successful ablation in patients with low-risk PTC. An advantage of applying radiomics in medical imaging is its ability to non-invasively extract data that is imperceptible by the human eye and thus not accessible through traditional visual inspection of the images. The implementation of radiomics on the post-treatment I-131 TBS has an advantage over other methods such as the use of pre-ablative pertechnetate scan because there is no need to perform additional procedures to the patients, and patients did not receive additional radiation. The radiomic processing time is acceptable to perform in clinical setting, however, a team of experienced personnels is still needed.

In attempting to explain the clinically relevant of the radiomic features for predicting successful ablation, we found that most of the significant radiomic features in our study were in the wavelet classes, which reflect the pixel intensity distribution, thus probably represent the tissue heterogeneity of the residual thyroid tissue. In brief, the increase in the tissue heterogeneity was associated with a failure of the ablation, as demonstrated by the example of the wavelet-HH_gldm_DependenceVariance feature.

**Figure 4:**
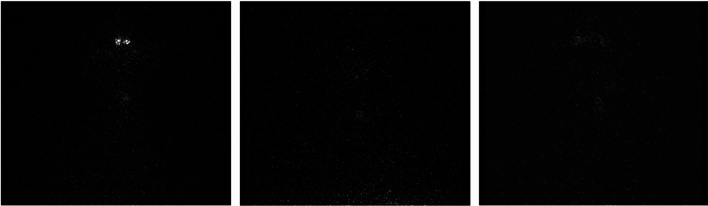
Three post-treatment static anterior thyroid images those are unable to define ROI by automated segmentation.

One of our strengths is that we used automated image segmentation to avoid intra- and inter-observer variability of the extracted radiomic features, which introduced less bias in the extracting process with high reproducibility^10,43^. Nevertheless, three patients were excluded due to the inability to perform automate segmentation because the intensity of the thyroid uptake was not clearly different from the background, as shown in Fig. 4. In this group, successful ablation was achieved in two patients (66.7%), while one patient did not meet the criteria of successful ablation (33.3%). The mean pre-ablative serum Tg level of those three patients was very low (0.48 ng/mL).

The limitations of our study were the retrospective nature and the lack of external datasets for the model validation. Therefore, further studies are needed to validate the model’s generalizability. Further investigation on the use of radiomic analysis on pre-treatment TBS to assess the probability of successful I-131 ablation in low to intermediate-risk PTC is probably helpful, because it would help determining individual activity of I-131 ablation/treatment, and thus allowed for personalized treatment. Other roles of TBS radiomics related to the prediction of disease recurrence, and disease-free survival could also be explored.

## Conclusions

Radiomic analysis of the post-treatment TBS combined with pre-ablative serum Tg showed a significant improvement in the predictive performance of successful ablation in low-risk PTC patients compared to the use of clinical parameter alone. Nevertheless, further validation in the larger external dataset is still required.

## Data Availability

The datasets generated during and/or analysed during the current study are available from the corresponding author on reasonable request.
